# Tinnitus, Suicide, and Suicidal Ideation: A Scoping Review of Primary Research

**DOI:** 10.3390/brainsci13101496

**Published:** 2023-10-23

**Authors:** Carol MacDonald, Charlotte Caimino, Georgina Burns-O’Connell, Douglas Hartley, Joanna Lockwood, Magdalena Sereda, William Whitmer, Rilana Cima, Laura Turton, Derek J. Hoare

**Affiliations:** 1 NIHR Nottingham Biomedical Research Centre, Nottingham NG1 5DU, UKderek.hoare@nottingham.ac.uk (D.J.H.); 2 Hearing Sciences, Mental Health and Clinical Neurosciences, School of Medicine, University of Nottingham, Nottingham NG7 2UH, UK; 3 Department of Psychology, University of Stirling, Stirling FK9 4LA, UK; 4 Nottingham University Hospitals NHS Trust, Nottingham NG7 2UH, UK; 5 Department of Audiology, Aston University, Birmingham B4 7ET, UK; 6 NIHR MindTech MedTech Co-Operative, Institute of Mental Health, Mental Health and Clinical Neurosciences, School of Medicine, University of Nottingham, Nottingham NG7 2UH, UK; joanna.lockwood@nottingham.ac.uk; 7 Hearing Sciences: Scottish Section, Glasgow, Division of Clinical Neuroscience, School of Medicine, University of Nottingham, Nottingham NG7 2UH, UK; bill.whitmer@nottingham.ac.uk; 8 Health Psychology, Faculty of Psychology and Educational Sciences, KU Leuven University, 3000 Leuven, Belgium; rilana.cima@kuleuven.be; 9 Tinnitus Center of Expertise, Centre of Expertise in Rehabilitation and Audiology, Adelante, 6432 CC Hoensbroek, The Netherlands; 10 Experimental Health Psychology, Faculty of Psychology and Neurosciences, Maastricht University, 6200 MD Maastricht, The Netherlands; 11 Audiology, NHS Tayside, Dundee DD3 8EA, UK

**Keywords:** tinnitus, suicidal thoughts, suicidal ideation, suicidal behaviour, suicide

## Abstract

Tinnitus (the perception of sound in the absence of any corresponding external source) is highly prevalent and can be distressing. There are unanswered questions about how tinnitus, suicidal thoughts, and suicidal behaviours co-occur and interact. To establish the extent of scientific literature, this scoping review catalogued primary reports addressing the associations between tinnitus, suicidal ideation, attempted suicide, and death by suicide. We searched OvidSP, Medline, EMBASE, PsycINFO, CINAHL, Google Scholar, EThoS, and ProQuest for all studies and case reports on ideation and/or attempted and/or completed suicide in the context of tinnitus. Twenty-three studies were included, and data were charted according to study type. Several epidemiological and other observational studies gave evidence of risk factors and an association between suicidal ideation, suicidal behaviour, and tinnitus. However, there was no evidence of the direction of causality. Qualitative studies are indicated to explore the patient’s experience and understand the dynamics of any interaction between tinnitus and suicidal thoughts and behaviours. A theory-informed model of tinnitus and suicide needs to be developed to inform the development of interventions and how tinnitus patients are supported clinically.

## 1. Introduction

Tinnitus, the perception of sound (e.g., ringing, humming, or hissing) in the ear(s) or head without corresponding external acoustic source, is a complex and heterogeneous condition. It is a common experience, with reported prevalence of 5.1% to 42.7%, depending on the population and definition of tinnitus used [1]. Whilst tinnitus is an unthreatening auditory sensation for some people, for others it can be distressing, causing significant emotional, behavioural, and functional difficulties, impaired quality-of-life, and adjustment and mental health problems [2]. Problematic tinnitus is managed across primary to tertiary care settings (General Practitioners, audiologists, ENT, psychologists, psychiatrists) [3]. The term Tinnitus Disorder has recently been proposed to distinguish tinnitus as a percept from tinnitus as a condition affecting health and quality of life [4]. 

A range of studies have identified the dynamic network interactions of emotional, cognitive, and behavioural factors which contribute to distress and disability experienced by people affected by tinnitus [5,6,7]. People with tinnitus and other co-occurring physical or mental health conditions may have a higher risk of expressing suicidal ideation and behaviour [8,9]. Suicide is a complex global problem which demands urgent action. More than 700,000 people die by suicide every year and for each adult who died by suicide there may have been more than 20 others attempting suicide [10]. Suicidal ideation may be defined as thoughts of ending one’s own life [11]. Generally, in suicide literature, multiple definitions and ways of measuring suicidal ideation are debated and used [11,12]. Traditionally, suicidal ideation and behaviour were theorised to exist and progress along a continuum of increasing risk from relatively passive thoughts to more active thoughts, plans, intent, and enaction [13,14]. This continuum model has been revised via Ideation to action theories and recent research to represent suicide risk as a non-linear dynamic process that can emerge and unfold over short windows of time, with multiple potential pathways between different suicidal thoughts and behaviours [15,16]. Suicidal ideation has been shown to be multidimensional, characterised by both passive (i.e., wish to die) and active components (i.e., desire to kill oneself) [16,17,18]. Suicidal ideation is one of the top three risk factors for death by suicide, and an important marker for clinical intervention and research investigation [19,20]. Suicidal ideation is highly prevalent worldwide, considerably more frequent than suicidal behaviour, and represents a much larger proportion of the population than suicide attempts, particularly within non-clinical samples [21]. However, incidence estimates vary depending on the definitions and wording used in different studies (ranging from thoughts that life is not worth living to making concrete plans to end one’s life). A study of World Mental Health Survey data from 17 countries reported that across lifetimes, 9.2% of the population will experience suicidal ideation, 3.1% will make suicidal plans, and 2.7% will attempt suicide [22]. It is estimated that 20.6% of people in the UK will experience suicidal thoughts in their lifetime [23]. Passive suicidal ideation is a common experience and associated with significant psychiatric comorbidity [17,23,24]. Passive and active ideation are highly correlated but distinct constructs that often co-occur and are similar in terms of psychiatric comorbidity, psychological characteristics, and associated risks [25]. Both may ebb and flow in a dynamic fashion toward potentiating suicidal behaviour, with the greatest risk occurring among individuals that report both passive and active ideation [26]. So far, the presence and contribution of suicidal ideation within biopsychosocial models of tinnitus has not been addressed.

Theoretical models of suicide seek to explain suicidal cognitions, behaviours, and the complex interaction between psychological, social, and physiological factors, and risk factors. Key dynamic and process models take a multifactorial perspective on suicidal behaviours and aim to understand the mechanisms of suicidal processes, especially the transition from suicidal thoughts to suicidal actions [27,28,29]. Mental illness tends to be foregrounded as a predominant risk factor for suicide but is not a sufficient marker of suicide risk, given that most people with diagnosable psychiatric illness will not die by suicide [30,31]. In addition to or beside mental illness, other factors are associated with the emergence of suicide risk. Many people die by suicide in moments of crisis, depleted in resources and the ability to cope with contextual stressors such as financial problems, relationship breakdowns, or health-related stressors caused by chronic conditions, such as chronic pain [32,33,34,35]. Medical conditions carry suicide risk, even when controlling for mental and substance use disorders [36,37]. As such, all chronic conditions including tinnitus should receive equitable attention in terms of suicide prevention care and research. 

The review literature on tinnitus and suicide is not extensive. A review by Jacobson and McCaslin et al. [38] included four studies and found “no evidence” of a predictive relationship between tinnitus and suicide without pre-existing psychiatric conditions. A more recent review by Szibor et al. [8] was limited in scope, omitting suicidal ideation, and concluded there was insufficient research evidence to determine the nature of any relationship between suicide and tinnitus. Tailor et al. [9] were the first to systematically review the prevalence of suicidal ideation among people with tinnitus. Their pooled data from six cross-sectional studies represented over 7000 people with tinnitus across four countries and gave an overall prevalence for suicidal ideation of 20.6% (95% CI, 10.8–30.3 per cent), which approximates to twice the prevalence seen in general populations. Key qualitative conclusions were that suicidal ideation may be more prevalent in tinnitus sufferers than in the general population, and more common in individuals with a higher degree of tinnitus-related distress. The study authors recommend the routine assessment of suicidal thoughts and behaviours in patients with tinnitus and, if suicidal ideation is present, formal suicide risk assessment and appropriate referral to mental health services. 

Previous reviews have appropriately limited inclusion of the literature on tinnitus and suicide to address specific questions. A comprehensive review of the field will provide insight by cataloguing the literature on tinnitus and suicide to identify (1) possible gaps in knowledge and (2) new studies and approaches to further understand the relationship between tinnitus and suicide and inform clinical practice. This scoping review aimed for comprehensiveness and the inclusion of all study designs, with an overarching focus on suicidal ideation, to catalogue what the primary literature can and cannot tell us about the association between tinnitus, suicidal ideation, and suicidal behaviour.

## 2. Materials and Methods

This scoping review was designed, conducted, and reported following the Joanna Briggs Institute guidance for conducting and reporting scoping reviews and the PRISMA Extension for Scoping Reviews (PRISMA-ScR) checklist and explanation [39,40].

### 2.1. Inclusion/Exclusion Criteria

Records were included where tinnitus was reported as the primary complaint, and suicidal ideation and/or attempted suicide and/or death by suicide were examined or reported. Records reporting both subjective tinnitus (i.e., only heard by the patient) and objective tinnitus (can be heard by an examiner) were eligible for inclusion. Eligible records reported randomised controlled trials, non-randomised controlled trials, cohort studies, case series, case studies, epidemiology articles, and sources reporting personal/expert opinions that included reference to actual cases. Records were included from all countries, providing they were available in English language. No exclusions by date were applied. Studies including both adults and children were eligible for inclusion. Review articles were not eligible. Records not available in English were excluded due to limited capacity for translation.

### 2.2. Information Sources

Literature searches were conducted in multiple databases using a tailored search strategy. The search strategy involved (1) searches of electronic databases of peer-reviewed journals using defined search terms, (2) searches of the grey literature, and (3) searches of the reference lists of included records. The online search interface OvidSP was used to search Medline, EMBASE, and PsycINFO databases and the search interface EBSCOhost was used to search the Cumulative Index to Nursing and Allied Health Literature (CINAHL). For searches of Google Scholar, a stopping rule was applied when three consecutive search pages retrieved no further relevant records. Theses were searched via EThoS and ProQuest online archives.

### 2.3. Search

We searched for records containing key terms “tinnitus” AND “suicide” OR “suicidal ideation”. The searches were expanded to capture alternative words and phrasing for each of the key terms. Search terms were tested and refined to ensure relevant and comprehensive results were retrieved. No limitations on study date were applied as all records relating to this topic were of interest. Initial searches were carried out in August 2019, updated over time, with a final update search conducted in Google Scholar in May 2023. Table 1 gives an example full search strategy in PubMed.

### 2.4. Selection of Sources of Evidence

Records identified from the electronic database and manual searches were downloaded with citation, title, and abstract, and imported into EndNote (v 20.2.1) reference manager. Duplicate records were removed. Initially, titles and abstracts were screened independently by pairs of reviewers (CC, CM, and DJH). All records selected by either reviewer were taken forward to full text screening. Full texts were screened independently by pairs of authors (CC, CM, and DJH) and any discrepancies in inclusion/exclusion decision resolved through discussion. 

### 2.5. Data Extraction and Charting

Data were extracted from the included full texts into a bespoke data charting form in Excel. The charting form was piloted on two records and the process and data items were discussed before commencing the full data extraction procedure. Data items included Author, Date, Study Title, Country, Study design, Population, Age (range and mean), Sample size, Participants with tinnitus (n), Type of tinnitus, Tinnitus severity, General method of the study, Methods used, Method/question used to identify tinnitus, Method/question used to identify bothersome tinnitus, Method/question used to identify suicide/suicidal ideation, Method of suicide, Comorbidities of suicide population, Main findings, Clinical recommendations, and Research recommendations. Four researchers (CM, CC, GBOC, and DJH) extracted the data from each record: two of four researchers independently extracted data and then compared it for accuracy. The accuracy of data charting was further verified in cross check by a third reviewer (CM or DJH). No critical appraisal of included sources of evidence was conducted. The data from Excel were then charted according to (1) study type, and (2) whether the record was concerned primarily with suicidal ideation, behaviours, or death by suicide.

### 2.6. Expert Consultation

The charted data and interpretation of the body of work were shared with peers who have expertise in tinnitus and/or suicide for comment and review of the initial interpretation. Experts were a practicing ENT consultant (DH), audiologist (LT), and clinical psychologist (RC) and researchers with expertise in tinnitus (RC, LT, MS, WW) and suicide/self-harm (JL). Feedback was used to refine the discussion to be a shared interpretation by all authors.

## 3. Results

**Figure 1:**
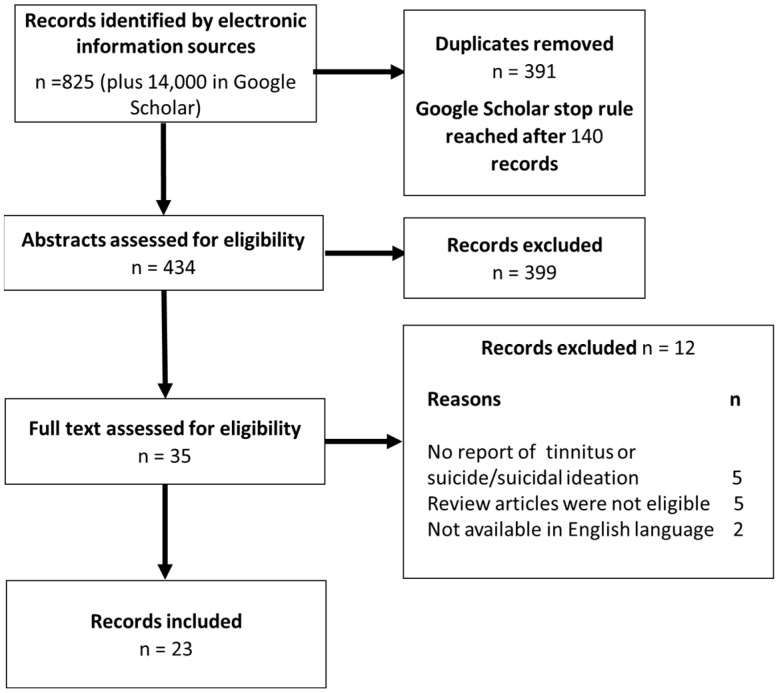
Flow chart of record screening.

Initial searches identified 825 records and over 14,000 in Google Scholar. Of the 35 full texts screened, 12 articles were excluded (5 did not report tinnitus or suicide/suicidal ideation, 5 were review articles, and 2 were not available in English language). This left 23 records eligible for the review (Figure 1). Two of these records were identified in update searches.

General characteristics of each record are presented in Tables S1–S3. Records were reported from the US (6), South Korea (6), UK (5), The Netherlands (2), and Brazil, China, Sweden, and Taiwan (1 from each). Included records were published across 1992–2023. We found notable variation in the use of terms to describe suicidal ideation and behaviours (e.g., “suicide wishes” [41], “unsuccessful suicide attempts… successful suicide attempts” [8], ”committed suicide” [42] “committing suicide” [43,44,45]. In reporting on the primary studies catalogued, terminology used by authors was retained to present their findings accurately.

### 3.1. Epidemiological Studies

Overall, 7 of the 23 included records were epidemiological studies (Table 2). Of these studies, five focused on suicidal ideation [44,45,46,47,48], one focused on both suicidal ideation and suicide attempts [43], and one focused on death by suicide [49].

#### 3.1.1. Suicidal Ideation

Five epidemiological studies published between 2018 and 2020 explored tinnitus and suicidal ideation in association with other variables and health conditions in the Korean population, using data from respondents to the South Korean national health and nutritional examination survey. Four records [44,45,46,48] studied adults, with sample sizes ranging between 4633 and 28,930. Of these, one study focussed on pre-menopausal women [44], and two studies focussed on adults aged over 50 and 60 [45,48]. There may be some overlap in samples between these studies which share common intranational features across a narrow recent timescale. One study [47] focussed on the adolescent population with a sample of 1587. Where studies focussed on specific sub-populations, samples were smaller. The number of participants with tinnitus identified in adult studies ranged from 934 [44] to 6391 [46]. The main difference in prevalence emerged when the focus turned to older respondents. Kim [47] identified 286 adolescents with tinnitus. All studies used the same single question to identify the presence of tinnitus and all assessed tinnitus severity using similar terms and grades, operationalising severity by degrees of annoyingness or bothersomeness and sleep interference. Four studies identified suicidal ideation by positive responses to a single question, asking participants if they had thought about committing suicide in the last 12 months. Han et al. [46] did not use the word “commit” but asked participants if they had seriously considered attempting suicide, with focus on identifying more severe ideation.

The findings and clinical recommendations of this group of studies were consistent. Han et al. [46] found a significant association of tinnitus with the presence of depressive mood and suicidal ideation. Of the 20.9% of the population sample with tinnitus, 18.8% participants had depression and 21.6% had suicidal ideation, compared to non-tinnitus participants with 12.3% depression and 13.1% suicidal ideation. It was found that this association increased with tinnitus severity: compared with adults with mild tinnitus, adults with severe tinnitus were 36% more likely to have suicidal ideation. Tinnitus, depressive mood, and suicidal ideation were shown to share common socioeconomic, stress, and health-related risk factors. Higher perceived stress was also associated with suicidal ideation, with the association of tinnitus with depression and suicidal ideation mediated by perceived stress. Mediation analysis suggested that tinnitus contributes to the development of depression or suicidal thoughts via an indirect pathway, with tinnitus playing a significant mediating role between depression and risk factors. Kim [47] found the prevalence of tinnitus to be 18% among the adolescent population. Tinnitus severity was shown to be directly associated with negative mental health outcomes and stress. Of adolescents reporting tinnitus, 21.5% also experienced suicidal ideation. This study concluded adolescents may struggle to understand and disclose their tinnitus and recommended (1) assessment and management of tinnitus in the development of interventions aimed at preventing or reducing depression and suicidal ideation in adolescents, and (2) specific intervention to prevent or reduce depression and suicidal ideation in adolescents with tinnitus.

Park et al. [48] investigated the relationship between tinnitus, joint pain, stress, depressed mood, and suicidal ideation. The incidences of stress, depressed mood, and suicidal ideation were found to be significantly higher in the tinnitus group. Suicide ideation was significantly higher in the tinnitus group than the non-tinnitus group (*p* < 0.001). The study showed that both the prevalence and severity of tinnitus were significantly related to joint pain and suggested that both conditions share psychological distress as a common risk factor. The authors recommended consideration of psychological distress as a risk factor for tinnitus and joint pain when deciding treatment strategies and in guiding public health policy but did not make recommendations with respect to suicide. Park et al. [45] found tinnitus to be a significant risk factor, both positively and independently associated with deteriorated mental health and health-related quality of life (QoL) in older adults. The percentage of participants reporting suicidal ideation in the ‘no tinnitus’ group was 4.8 ± 0.5, in the ‘tolerable tinnitus’ group was 9.8 ± 1.5, and in the ‘annoying tinnitus’ group was 10.9 ± 1.6 (*p* < 0.001). The authors recommended comprehensive care for improving mental health and QoL when treating older tinnitus patients, especially those with annoying tinnitus. Again, suggestions were posited within a mental health context as opposed to specific recommendations for suicide prevention. Yu et al. [44] found that women with tinnitus had significantly higher rates of stress, depressive mood, and suicidal ideation than those without tinnitus (*p* < 0.001). The authors discussed the clinical implications of menstrual cycle irregularity in assessing tinnitus in pre-menopausal women, and appropriate interventions to alleviate tinnitus symptoms through accurate assessment and management of menstrual cycle irregularity. Implications with respect to suicide were not discussed.

#### 3.1.2. Suicidal Ideation and Behaviour

A landmark study by Seo et al. [43] analysed the relationship between tinnitus, suicidal ideation, and suicidal behaviour using data from a sample of 17,446 Korean men and women aged 19 years and older. The mean age of participants was 48.3 years. The presence of tinnitus was identified in 3949 (21.4%) participants and tinnitus severity was categorised as not annoying/annoying; irritating; severely annoying and causes sleep problems. A total of 20.9% and 1.2% of participants with tinnitus, and 12.2% and 0.6% of those without, reported suicidal ideation and attempts, respectively (*p* < 0.0001 and *p* = 0.001). Participants reporting suicide attempts showed a higher proportion of severe annoying (6.0%) and irritating (11.8%) tinnitus than those with suicidal ideation (1.4% and 10.2%, respectively). The study found that risks for experiencing tinnitus were significantly associated with suicidal ideation and attempts after adjusting for confounding variables. The prevalence of suicidal ideation and attempts in participants with tinnitus was significantly higher than among those without tinnitus after adjusting for sociodemographic factors and comorbidities. The authors conclude that their findings, the first evidence for this association in a large-scale study, have important implications for enhanced screening and evaluation of mental health status and suicidal ideation and behaviour among tinnitus patients, and the key role of physicians in management and care.

#### 3.1.3. Death by Suicide

In the only large-scale study of death by suicide, Martz et al. [49] conducted a retrospective analysis of the prevalence of tinnitus, depression, anxiety, and suicide in a population of recent veterans in the USA. In total, 769,934 veterans aged under 26 years or over 50 years who sought medical aid from the Veterans Administration health care system between January 2002 and December 2011 and who were not killed in action were included. Subjective tinnitus was diagnosed in 116,358 (15%) of the sample. Of the veterans diagnosed with tinnitus, 21% had reported symptoms of depression, 8% anxiety, and 17% reported having both. Of veterans with tinnitus, 54% did not report depression or anxiety. Counter to the initial hypothesis, the study found that suicide rates were lower among veterans with tinnitus than veterans without tinnitus, and that co-occurring diagnoses of mental health conditions did not significantly increase the risk of suicide. Discussing their counterintuitive findings, the authors speculated that coping strategies for tinnitus may have protected some veterans against suicide. Nonetheless, health care professionals, such as audiologists and psychologists, should be aware of the associations between tinnitus and mental health problems, prepared to address the psychological needs of individuals who have tinnitus; those with tinnitus and co-occurring depression and anxiety should receive clinical care to help lower their symptoms and raise their quality of life. The authors challenge the automatic assumption that “tinnitus may lead to suicide” and suggest that the strength of negative psychological states cannot be predicted solely by the onset of chronic impairment or disability in this population. 

### 3.2. Case Studies

Twelve records reported cases of co-occurring tinnitus and suicidal thoughts and behaviour, including death by suicide [41,42,50,51,52,53,54,55,56,57,58,59] (Table 3). Five studies came from the USA [52,54,55,58,59], three came from the UK [42,56,57] two from the Netherlands [41,53], one from Taiwan [50], and one from Brazil [51].

#### 3.2.1. Suicidal Ideation

Three studies presented the application of successful tinnitus treatment in single cases of severe tinnitus and self-reported suicidal ideation in an adult female, a 69-year-old male, and an 11-year-old boy [41,50,51]. A striking case series by Fox-Thomas [52] studied 200 adult tinnitus patients aged from 25–79 years who completed the Tinnitus Reaction Questionnaire (TRQ [60]). Of the sample, 59% were male and 41% were female. The average total score on the TRQ was 72 (in the “profound” category) but one quarter of the sample scored at mild or moderate tinnitus. Suicidal ideation was identified in 32 patients (16% of the sample) by a score of at least 1 to item 24 on the TRQ: “My tinnitus has led me to think about suicide”. Most patients (75%) reported thinking about suicide “a little” or “some” of the time. One in four patients (25%) reported thinking about suicide “a good deal” or “almost all” of the time. Almost half of the patients with profound tinnitus disturbance reported thinking about suicide. However, a key finding was that suicidal thoughts were reported by some patients with mild (5%) or moderate (20%) tinnitus disturbance. Notably, the study found that the prevalence of suicidal ideation was four times greater among this sample of patients with tinnitus than in the general US population with other chronic health conditions, except for chronic pain.

#### 3.2.2. Suicidal Behaviour

A series of four cases [54] and three single case studies [53,55,59] described attempted suicide by three females aged between 41 and 80+, and four males aged between 40 and 80+ with tinnitus. These studies presented the challenge of tinnitus treatment in the context of interactions between medication, pre-existing and worsening mood disorders, and tinnitus severity. All recommend that clinicians should comprehend the impact that tinnitus can have on their patients’ lives and select treatments accordingly, especially for those with a history of mood disorders. A narrowly focused prevalence study of tinnitus and attempted suicide by overdose [56] found just three patients with tinnitus who had been admitted to the poisons unit of a single UK hospital in a 3-month period. Compared to rates of tinnitus in the general population (around 7%), the prevalence of tinnitus in this population sample was unexpectedly low (1.6%). None of the three patients stated that tinnitus had influenced their decision to take an overdose, which the authors described as parasuicide. These findings were attributed to either well-developed local services for providing help to those with tinnitus, or that tinnitus patients were harming themselves in ways other than self-poisoning.

#### 3.2.3. Death by Suicide

Two studies examined death by suicide (a low base rate event) in a retrospective survey of 28 cases from 50 audiology clinics worldwide [42] and in 6 out of 674 tinnitus patients in Cardiff, Wales [57]. These studies identified common risk factors. The Cardiff study found a high rate of suicide among their clinical population (a rate of 118 per 100,000 per year), compared with the general population rate in the wider county (9 per 100,000 per year). The six patients who died had severe subjective tinnitus; their ages ranged from 33–76 years (mean = 58 years). The authors discuss the difficulty of ascertaining causality and representative accuracy concerning rates of suicide in people with tinnitus based on this limited number of cases. Nonetheless, tinnitus severity appeared to have been a significant factor in these reported cases of death by suicide. Common risk factors for suicide were identified and discussed: patients were mainly working class, male, socially isolated and living alone, and had experienced bereavement and psychological problems. The most reported mental health problem was depression, common to all six individuals. Lewis, Stephens, and McKenna [42] analysed data from their retrospective survey of cases of twenty males and eight females with tinnitus who died by suicide; their ages ranged from 17 to 82 years (mean = 57.1 years). Male gender, being older than 50 years, and social isolation were identified as common risk factors for suicide. Psychiatric symptoms at time of death were present in 95% of subjects, with depressive symptoms in 70% and “alcoholism” in 17%. A suicide attempt within the last year was identified as a key risk factor. Tinnitus had been present less than 1 year in 40% of deaths by suicide, compared with around 15% of patients attending audiology clinics, indicating tinnitus as a significant factor in death by suicide within a short time of tinnitus onset. The authors suggest that whilst it is unlikely for tinnitus alone to cause suicide, it should be considered a predisposing factor interacting with others, notably the variables they identified which raise risk of suicide. By contrast, Pridmore, Walter, and Friedland [58] presented a case series of four males with tinnitus aged between 29 and 66 who died by suicide with no diagnoses of mental health disorders recorded. Each case had been assessed by a coroner in the last 10 years, and the accounts were public records, found in newspaper and online articles from the USA, Belgium, and the UK. The article detailed names and methods of suicide. The authors acknowledge the limitations of their small and select series of cases in offering evidence for or against a cause-and-effect relationship between tinnitus and suicide. However, they suggest that it would be perverse to attribute suicide to depression in all cases like this without compelling evidence, and that in rare cases, highly distressing precipitants alone may be enough. The authors emphasised the need for compassionate clinical care for significantly distressed people for whom no method of treatment for their tinnitus may be successful, and to be ready to refer those with severe, disabling tinnitus to a psychiatrist or another mental health professional for diagnosis and treatment of any underlying psychiatric disorder.

### 3.3. Observational Studies

Of five observational studies, two cross-sectional studies came from the same UK tinnitus clinic [61,62], one came from China [63], one from Sweden [64], and one from Taiwan [65] (Table 4).

#### 3.3.1. Suicidal Ideation

Three included records used anxiety and depression questionnaires to study suicidal ideation in patients aged between 16–95 years at a UK tinnitus clinic [61,62], and 11–86 years in an outpatient department in China [63]. Aazh and Moore [61] conducted a correlational study of thoughts about suicide and self-harm in 150 out of 402 patients who completed survey questionnaires. Thoughts of suicide and self-harm were measured by responses to question 9 of the Patient Health Questionnaire (PHQ9 [66]): “Over the last two weeks, how often have you been bothered by thoughts that you would be better off dead or of hurting yourself in some way? (Not at all, several days, more than half the days, nearly every day)”. The study found that 13% of patients reported self-harm or suicidal ideation in the 2 weeks prior to clinical assessment. Self-harm and/or suicidal ideations were moderately correlated with anxiety and depression scores, as measured using the Hospital Anxiety and Depression Scale [67]. Only small correlations were found between self-harm and suicidal ideation and tinnitus handicap (r = 0.21) and insomnia (r = 0.20). Correlations were slightly higher between anxiety (r = 0.35) and depression (r = 0.31) and self-harm and suicidal ideation. Aazh et al. [62] conducted a retrospective cross-sectional study to explore whether parental mental illness in childhood is a risk factor for suicidal and self-harm ideations in 292 adults who sought help for tinnitus and/or hyperacusis. Of the 292 patients, 286 had mild, moderate, or severe tinnitus. As in the previous study [61], thoughts of suicide and self-harm were measured by responses to question 9 of the PHQ9. Forty percent of patients had depression, and 45% had anxiety. The study found that 46 of 292 patients (15.75%) had experienced suicidal and self-harm ideations within 2 weeks prior to assessment. In addition, 38.7% of patients reported a history of parental mental illness during childhood. Two variables (a childhood history of parental mental illness and current depression level) were associated with the risk of suicidal and self-harm ideations in this clinical sample. Both studies recommend clinicians be alert to the possibility of suicide and/or self-harm ideation in tinnitus patients and to screen and refer people for appropriate mental health treatment, especially those with symptoms of depression and a childhood history of parental mental illness.

In a cross-sectional cohort study, Chen et al. [63] used network analysis to examine the comorbidity of depression and anxiety symptoms in 566 people with tinnitus recruited at a tinnitus outpatient department in China. Network analysis using items from anxiety and depression questionnaires highlighted central and bridge symptoms within the interacting network of depression and anxiety in the sample of tinnitus patients. Analysis showed a direct relation between D9 “Suicidal ideation” and A7 “Afraid something awful might happen” was the strongest, followed by D2 “Feeling depressed or hopeless” in this sample. The authors recommend that clinical prevention and psychotherapy should be implemented to target the symptoms with the strongest associations with suicidal ideation, and to reduce anxiety symptoms.

#### 3.3.2. Suicidal Behaviour

Two studies reported an association between tinnitus and attempted suicide. Lugo et al. [64] assessed the sex-specific association of tinnitus with suicide attempts in a cohort study of 71,542 (31,545 male, 39,997 female) adults who participated in the 2010 Stockholm Public Health Cohort survey. Suicide attempt(s) were reported by 2404 (3.4%) respondents. The risk of suicide attempt was 15% higher than average in those who had tinnitus, 32% higher in those who have severe tinnitus, not higher in those who had received a tinnitus diagnosis, and higher in women who reported severe tinnitus. Thus, the study found a sex-dependent association of tinnitus with suicide attempts, with severe tinnitus associated with suicide attempts in women but not in men. An interesting finding was that participants who had been diagnosed with (and possibly treated for) tinnitus were not at increased risk of suicide, suggesting a protective function of clinical care for patients with tinnitus. As only one fifth of participants with severe tinnitus were diagnosed by a specialist, the authors identified unmet clinical need and recommended increased resource for the management of tinnitus in clinical practice.

Using data from Taiwan’s National Health Insurance Research Database, Cheng et al. [65] followed up 386,055 tinnitus patients aged ≥20 years who received a first-time diagnosis of subjective tinnitus and had no history of suicide for 3 years before tinnitus onset. Tinnitus severity was not reported. A comparison cohort of 386,055 matched controls with no tinnitus was established. Sampled patients were tracked during 1-year-follow-up to identify any claim with a diagnosis of suicide attempt as indicated in ICD codes. Deaths by suicide were classified as suicide attempts. The results show that the incidence of attempted suicide was 0.253 (95% CI = 0.237–0.269) and 0.123 (95% CI = 0.113–0.135) for the study cohort and comparison cohort, respectively. A log-rank test suggested the study cohort had significantly lower suicide attempt-free survival at 1 year than the comparison cohort (*p* < 0.001). Thus, it was found that the study cohort had a higher hazard of suicide attempt, with an incidence of attempted suicide 2.06 times higher than that of controls. Cheng et al. [65] recommended clinicians and healthcare providers to be aware of the potential role of tinnitus in suicidal behaviour, to assess individuals with tinnitus for suicidality and to refer for comprehensive mental health evaluation when appropriate.

### 3.4. Research Recommendations in the Included Records

Research recommendations in single case studies reflected the specific treatment benefits and risks reported, and the limits of *n* = 1 studies. Chang and Wu [50] called for controlled trials of duloxetine in patients with tinnitus to substantiate their account of Mr. A’s recovery from symptoms of both tinnitus and depression. Ensink et al. [41] emphasised the need for long term follow-up with patients treated with eustachian tube blockage to evaluate long term effect of treatment. The findings of the self-poisoning case series by Lewis and Stephens [56] prompted research in areas where tinnitus services are underdeveloped.

Several observational studies and two epidemiological studies recommended further research into genetic and predisposing risk factors for tinnitus and suicide. Aazh et al. [62] suggested examining the relationship between a broader range of adverse childhood experiences with suicidal and self-harm ideations among patients with tinnitus and hyperacusis. Lugo et al. [64] recommended research to understand the pathophysiological differences between men and women with tinnitus. Han et al. [46] recommended further research into neurobiological mechanisms of stress and tinnitus. Yu et al. [44] proposed the study of genetic factors which potentially contribute to the association between menstrual cycle irregularity and tinnitus.

Several epidemiological studies also recommended research to design and use better assessments and measurements. Kim [47] proposed developing assessment and measurement tools for both physical and psychological health status and tinnitus in young people. Han et al. [46] recommended the use of validated scales for assessment of tinnitus, depression, and suicidal ideation rather than survey questionnaires in future epidemiological studies. The limitations of questionnaire-based data and cross-sectional designs were discussed in many records [44,45,46,47,48]. To clarify the direction of causality and the relationship in the association between variables, researchers call for prospective studies and randomised trials [47], longitudinal studies [46,67], prospective studies with a long follow-up period [45], and case-control and cohort studies [44] on suicide and tinnitus. Martz et al. [49] discuss the complexity of suicide risk and suggest that a multidimensional approach should be used when researching suicide.

Cheng et al. [65] identified that their use of ICD codes from a healthcare claims database limited information about suicidal ideation, death by suicide, and relevant clinical information such as tinnitus severity, duration and treatment, and socioeconomic and personal factors. They recommended additional clinical studies to confirm the findings of their study and to increase understanding of this issue to support the development and application of optimum interventions for tinnitus and accessible mental health services to reduce suicide attempts in this population. Fox-Thomas [52] recommended further research to identify important warning signs and factors (problems such as depression, sleep disturbance, sound sensitivity) which may contribute to suicidal ideation among tinnitus patients. Finally, Chen et al. [63] recommended investigations to evaluate the potential influence of depressive and anxiety symptoms on the network estimated in their study and identify the need for a large sample of psychological data.

## 4. Discussion

The aims of this scoping review were to catalogue the literature on tinnitus and suicide, to identify gaps in the literature, unanswered questions, and opportunities to undertake new studies to further understand the relationship between tinnitus and suicide that will inform clinical practice. We found suicide ideation reported to be a common experience for people with tinnitus, which highlights the need for it to be integral to clinical practice. Shared risk factors and distress/stress levels were found for both tinnitus and suicidal ideation. This supports future study of transdiagnostic psychological factors in co-occurring tinnitus and suicidal ideation.

Most of the included records on tinnitus and suicide were quantitative cross-sectional or retrospective studies of the presence or severity of tinnitus, suicidal thoughts/behaviour, and co-occurring mental health disorders. They focused on the between-person characteristics which mark those who experience suicidal ideation and behaviours and tinnitus. Individual cases and case series had different emphases: some warned of extreme and exceptional cases related to acute physical and /or psychological presentations and treatment. A few individual case studies and series offered details of the sequential pattern and interactions between tinnitus and suicide ideation and behaviour, but at some distance from these people’s stories and lives. As no qualitative studies were found, we can highlight the complete absence of patients’ voices in the literature. Almost none of the case studies emphasised the impact of factors in the context of people’s lives and their experience of tinnitus, not just mental health diagnoses. It is important to understand the interaction between tinnitus, suicidal ideation, and behaviour as an emergent process that evolves within individuals over time. By understanding the dynamics of suicidal thinking, and connecting suicide theory to the experiences of individuals, we might gain new insights into the mechanisms through which suicidal thoughts and behaviours develop in relation to tinnitus, which will usefully inform clinical practice.

### 4.1. Associations between Tinnitus and Suicide and Other Factors

Across studies, variation in reported prevalence was due to sample variation in sociodemographic factors, comorbidity, psychiatric illness, and differences in methods, assessments, and measures for tinnitus and suicidal ideation and behaviour. There was mixed evidence for an effect of gender. There are known gender differences in lethality of suicidal behaviour and cultural patterns (methods and access to means); the rate of suicide attempts by men is lower and the rate of deaths by suicide is higher than in women [11,22]. However, in the current review, rates of suicidal ideation or behaviour were reported more often to be higher in females than males. Evidence that age group is a risk factor for tinnitus and suicidal ideation or behaviours was also mixed. There was some evidence for an association between the severity of tinnitus with depressive mood and suicidal ideation, e.g., Han et al. [46] found a higher prevalence of suicidal ideation with increasing tinnitus severity. However, they were unable to comment further on the underlying association and potential moderating or mediating factors between tinnitus severity and suicidal ideation due to a lack of data on temporality of symptom onset and limited data on co-occurring psychiatric illness. In a smaller sample, however, Fox-Thomas [52] identified suicidal ideation in cases of tinnitus which had been assessed as mild. It is important to note that depression does not differentiate between individuals with suicidal ideation and those who make attempts [68]. Suicide is a behaviour not a diagnosis, and current thinking in suicidality directs future research to think beyond mental health diagnostic categories and overreliance on formal psychopathology, comorbidity (especially depression), formal psychiatric risk assessment, and stratification [18,69]. We need to examine factors common to tinnitus distress and suicidal ideation and behaviours through the prism of pre-existing dynamic and psychological models (e.g., the integrated motivational-volitional model of suicidal behaviour [29], the interpersonal theory of suicide [70]) to understand the direction and interrelationships in the association. This would have the immense value of not only extending our understanding of the relationship between suicide and tinnitus, but also afford the opportunity to add to those existing models. This approach has been proposed for studying the relationship between chronic pain and suicide [71]. Internal entrapment, defeat, impaired positive future thinking, external entrapment, perceived burdensomeness, thwarted belongingness, and mental imagery have been shown to be important psychological factors for current suicide ideation [72]. Models of tinnitus and research studies have mapped out interactions between factors such as worry, threat perceptions, depressed mood, hopelessness, and loss of control in tinnitus-related distress [73,74]. The contextual impact of altered sense of identity as well as interference in and interruptions to living are relevant factors for study [75]. Future research could focus on the function of suicidal ideation within individuals’ experiences of tinnitus, upon protective psychological and social factors which may buffer risk and progression to suicide, the positive and negative factors in coping, and the impact of these factors upon tinnitus severity and distress.

### 4.2. Suicidal Ideation as a Predictor of Risk

Although suicide attempts are often considered among the strongest univariate predictors of suicide death, meta-analytic data suggest that suicide ideation confers comparable levels of risk [76]. Considering the prevalence of suicidal ideation relative to suicidal behaviour, advancing our ability to identify suicidal ideation is clinically relevant. However, there is very little literature that can inform how this is undertaken, and performed well, in tinnitus populations. The role of suicidal ideation is scarcely mentioned in cognitive behavioural frameworks of tinnitus and underexplored in records in the current review. More detailed understanding of how people understand their own experience of tinnitus, suicidal thoughts, and behaviours and how they interact, and how individuals perceive the role and status of tinnitus in their loss of secure connection to life, is clearly needed. Suicide is a psychological process and preventable. Incorporation of current theories, models, and studies of suicide would benefit tinnitus research as well as clinical practice. Tailor et al. [9] argue that persistent suicidal ideation may protect against death by suicide. However, current studies identify the complexity of risk [22,25,26,77], and have updated our understanding of the constructs of passive and active suicidal ideation and why assessment and treatment of both is good practice; the Fluid Vulnerability Theory of Suicide [78] conceptualises suicide as an inherently dynamic construct that follows a nonlinear time course and differs drastically within an individual over time. This theory posits that each individual exhibits a differential baseline risk for suicidal thoughts and behaviours as a function of individual diatheses [79,80]. Suicidal behaviours are heterogeneous, resulting from complex interactions between many different risk factors and symptoms [81]. Fluctuating internal or external risk factors and the relations between them can be better understood via network perspectives [78], dynamic study e.g., [82], and life-course models of suicide (e.g., ideation-to-action, stress-diathesis). Suicidologists acknowledge the limits of modelling this complexity (discussed as “equifinality”) whilst emphasising the need for qualitative research to represent people and gain richer descriptions of suicidal phenomena; such insights are needed to develop quantitative assessments with well-defined outcomes and more precise measurement [18]. Thus, it remains valid to explore factors in suicidal processes within specific subpopulations (e.g., the experiences of people struggling to live with tinnitus) and to take a micro-longitudinal approach [16].

That there is some association between tinnitus and suicidal ideation and behaviours was evident in this review, but this was established via de-contextualised and repeated cross-sectional studies of large population studies and datasets, which were inevitably unable to identify clear causal relationships. In some case studies where people died by suicide, methodological problems relating to accuracy of representation and retrospective analysis constrained interpretation of findings.

### 4.3. Suicide Nomenclature in Tinnitus Studies

The challenge of nomenclature is observed in this review and across suicide research more generally and remains critical in suicide prevention and clinical management [12,81]. Table 5 summarises a recent consensus on definitions for common terms. The tasks of reducing stigma, limiting the use of negatively associated language (e.g., “committed”, “failed attempt”, “suicide gesture”, and using language that accurately and sensitively describes experience (e.g., “died by suicide”) are also relevant for tinnitus research and care [83,84,85,86].

The literature showed variation in terms designating nonfatal suicidal behaviour. The use of the terms “parasuicide” and “overdose” in the case series by Lewis and Stephens [56] to report the self-poisoning of three patients reflects the historical context. These incidents were not thought to be serious when assessed on conventional criteria by the liaison psychiatrist. Then, the term “parasuicide” signified a non-fatal act in which an individual deliberately causes self-injury and/or takes a substance in excess of any prescribed or generally recognised therapeutic dose, irrespective of the purpose or motivation underlying the act [87]. However, given associated dismissive connotations and improved understanding of the continuity between self-harm and suicide [88], use of the term “parasuicide” has been discontinued. There is evidence for the weak relationship between the suicidal intent and lethality of a suicide attempt [79,89], and distress can be intense and high-risk even when medical consequences may be assessed as non-severe and dismissed [90]. Currently, the term “non-suicidal self-injury” is used in the USA whereas the term “self-harm” is used in the UK, in accordance with UK national clinical guidance, to refer to any act of self-poisoning or self-injury irrespective of the apparent motivation [91,92]. It is not easy or judicious to categorise self-harm as either suicidal or non-suicidal, as within and between episodes, it can be both and reflect mixed and changing motivations over time [76,88]. Likewise, perceived ‘desire to die’ connected to self-harm episodes is also transient and fluctuating. The likelihood of non-fatal repetition of self-harm and of suicide is raised after self-harm in adults: 1.6% of people seen in hospital after self-harm have been estimated to die by suicide in the next 12 months [93]. Self-harm is a predictor of suicide attempt or death by suicide [94]. Thus, all self-harm and suicidal thoughts and behaviours need to be considered in the context of tinnitus care.

### 4.4. Issues with Diagnoses and Treatment in Research Studies

Some studies in this review assumed mental health pre-morbidity or co-morbidity, especially when largely reliant on medical health records and brief measurements of various variables that may have minimised tinnitus as a factor. Case studies excluded patients’ and families’ accounts, and some studies focused on a narrow range of services attended, potentially missing many people who may have been experiencing suicidal ideation. Pridmore et al. [58] was a notable exception as coroners’ reports gathered evidence from families.

For future research, we recommend incorporating methods such as single case experimental design and ecological momentary assessment, network analysis, and longitudinal studies to represent patient experiences of co-occurring tinnitus and suicide e.g., [63,95]. Guidelines on ethical retrospective analysis and reporting of people’s deaths by suicide should inform future research and description of specific methods of self-harm and suicide should be avoided. Likewise, the literature on psychological autopsy and the misrepresentation of mental health problems should be considered in analysis and reporting.

We found wide variation in the methods and questions used to assess the presence and severity of tinnitus and suicidal ideations and behaviours. The definition and measurement of suicidal ideation and behaviours were not consistent across studies (a persistent problem of suicide research). Measures and questions used in included studies varied widely in between-measurement intervals, frequency, use of terms, and presence and absence of distinctions between self-harm, passive and active ideation, and planning with intent. Given the differences in terminology, measurement, and timescale, study samples contained people with widely differing levels of tinnitus distress, suicidal intent and ideation, and outcomes. Suicidal ideation and its risk factors often vary considerably over a period as short as 4–8 h [96] and commonly used measures do not capture variability. Item 9 of the PHQ9 evaluates passive thoughts of death or self-injury within the last 2 weeks and is often used to screen depressed patients for suicide risk. The ambivalence of this item has been discussed in previous studies [97,98]. The PHQ9 is recommended by NICE guidelines for mental health and tinnitus [99,100] and was used by three studies in this review. In one of those studies by Chen et al. [63], self-harm was not distinguished from suicidal ideation in their interpretation of PHQ-9 item 9. More sensitive measurement of both suicidal ideation/behaviours and tinnitus across time could improve understanding of the interactions.

Recent studies have demonstrated how frequent measurement of suicidal ideation can support patients [101,102]. The use of repeated measures which assess the psychological impact of tinnitus and which ask clear questions about frequency of suicidal ideation and behaviour may be particularly effective in research and in healthcare settings. Indeed, the British Society of Audiology practice guidance for tinnitus in adults [103] recommends standard use of the Clinical Outcomes in Routine Evaluation-Outcome Measure (CORE-OM [104] in tinnitus care. The factors which influence disclosure of self-harm and suicidal ideation and behaviour were not discussed in the literature, and there was scant detail of clinicians’ practices and perspectives, and patient perspectives. A recent study has suggested that between 50–60% of people who had experienced suicidal ideation or behaviour had not disclosed this to anyone else [105]. We do not know how consistently or effectively suicidal thoughts and behaviours are assessed and measured in clinical services for tinnitus. However, several studies included in our review indicate the value of early diagnosis and support, and studies by Lugo et al. [64] and Martz et al. [49] offered some evidence that clinical contact may be protective. Therefore, we recommend further study into the facilitators and barriers to effective assessment and mitigation of suicidal ideation and behaviours to inform the collaborative process between clinicians and tinnitus patients.

Overall, studies offered little or no detail of treatment for suicidal ideation and behaviours and co-occurring tinnitus. We found vague recommendations for psychological support and a lack of specific attention to suicide prevention in tinnitus care. Many of the studies in this review preceded EU, BSA, and NICE recommendations and practice guidelines [100,103,106]. Clinical recommendations for alertness and attention were made in several studies but were not operationalised. There is scope for more focussed discussion of suicide prevention in audiology, and many questions remain concerning the clinical management of suicide risk in people with tinnitus. Tailor et al. [9] recommended asking about suicide as standard in audiology but with a focus on stratification of risk rather than psychosocial assessment and mitigation. Effective and compassionate clinical mitigation could support both research and patients in clinic, and thereby strengthen the protective function of clinical contact—a finding of this review. Early identification of suicidal ideation, the vast and hidden part of the “iceberg” of suicidal processes, could be explored as a potential means to lower the burden, severity, and chronicity of tinnitus distress, and work to prevent suicide [14].

## 5. Conclusions

This scoping review indicates the value of further study of the issues and unanswered questions discussed. We have identified the need for a theory-informed model of tinnitus and suicidal ideation and behaviours. A qualitative, directional understanding of the interaction between tinnitus and suicidal thoughts and behaviours is lacking and much needed. Qualitative studies to understand people’s unique dynamic experiences of suicidal ideation and behaviours within tinnitus, across time and by their own account, are an important next step. Increased understanding will inform clinical guidance, training, and management, and the future work needed to advance clinical practice.

## Figures and Tables

**Table 1:** Example of advanced search terms in Pubmed.

Search: **((tinnitus) AND (suicide)) OR (suicidal ideation)** ((“tinnitus”[MeSH Terms] OR “tinnitus”[All Fields]) AND (“suicid”[All Fields] OR “suicidal ideation”[MeSH Terms] OR (“suicidal”[All Fields] AND “ideation”[All Fields]) OR “suicidal ideation”[All Fields] OR “suicidality”[All Fields] OR “suicidal”[All Fields] OR “suicidally”[All Fields] OR “suicidals”[All Fields] OR “suicide”[MeSH Terms] OR “suicide”[All Fields] OR “suicides”[All Fields] OR “suicide s”[All Fields] OR “suicided”[All Fields] OR “suiciders”[All Fields])) OR (“suicidal ideation”[MeSH Terms] OR (“suicidal”[All Fields] AND “ideation”[All Fields]) OR “suicidal ideation”[All Fields]) **Translations** **tinnitus:** “tinnitus”[MeSH Terms] OR “tinnitus”[All Fields] **suicide:** “suicid”[All Fields] OR “suicidal ideation”[MeSH Terms] OR (“suicidal”[All Fields] AND “ideation”[All Fields]) OR “suicidal ideation”[All Fields] OR “suicidality”[All Fields] OR “suicidal”[All Fields] OR “suicidally”[All Fields] OR “suicidals”[All Fields] OR “suicide”[MeSH Terms] OR “suicide”[All Fields] OR “suicides”[All Fields] OR “suicide’s”[All Fields] OR “suicided”[All Fields] OR “suiciders”[All Fields] **suicidal ideation:** “suicidal ideation”[MeSH Terms] OR (“suicidal”[All Fields] AND “ideation”[All Fields]) OR “suicidal ideation”[All Fields]

**Table 2:** Epidemiological studies.

Author	Date	Title	Country	Population	Sample Size	Participants with Tinnitus (n)	Tinnitus Severity	Method/Question Used to Identify Tinnitus	Method/Question Used to Identify Bothersome Tinnitus	Method/Question Used to Identify Suicide/Suicidal Ideation	
Han et al. [46]	2018	Tinnitus, depression, and suicidal ideation in adults: A nationally representative general population sample	Korea	Respondents of tinnitus evaluation questionnaires of the KNHANES aged 19+	28,930	6391	Mild, moderate, and severe (no disturbance, annoying and very annoying)	During the past year, did you ever hear a sound (buzzing, hissing, ringing, humming, roaring, machinery noise) originating in your ear? (yes, no, cannot remember)	How much did the sound originating in your ear disturb your daily life? (no, annoying, very annoying level causing sleep disturbance).	Within the past year, have you ever seriously considered attempting suicide? (yes or no)	No suicide—ideation reported
Martz et al. [49]	2018	Tinnitus, Depression, Anxiety, and Suicide in Recent Veterans: A Retrospective Analysis	USA	Veterans who separated from service and who were not killed in action.	769,934	116,358 (15%)	Not reported	NA-diagnosed with tinnitus	Not reported	Not reported	ICD-10 codes were used to identify cause of death.
Kim et al. [47]	2018	Association between tinnitus and mental health among Korean adolescents: The Korea National Health and Nutrition Examination Survey	Korea	Adolescent respondents of the KNHANES V	1587	286	No problem, bothering, having trouble sleeping	In the past 12 months, have you been bothered by buzzing in your ears? (yes, no)	How much of a problem is the ringing in your ears? (no problem, bothering, having trouble sleeping)	Have you ever thought of committing suicide in the last year? (yes, no)	No suicide-ideation reported
Seo et al. [43]	2015	Relationship between tinnitus and suicidal behaviour in Korean men and women: a cross-sectional study	Korea	Korean individuals who participated in 2010 to 2012 Korean National Health and Nutrition Examination Survey (KNHANES)	17,446	3949 21.4%	Not annoying/annoying; irritating; severely annoying and causes sleep problems	Within the past year, did you ever hear a sound (buzzing, hissing, ringing, humming, roaring, machinery noise) originating in your ear?	How severe is this noise in your daily life?	In the last 12 months, did you think about committing suicide?	NA
Park et al. [48]	2020	Psychiatric Distress as a Common Risk Factor for Tinnitus and Joint Pain: A National Population-Based Survey	Korea	General over 50s	9032	2413	Classified as none, not annoying, annoying, and severely annoying	Single question	Single question: none, not annoying, annoying, severely annoying.	Positive answer to the question about suicidal ideation and suicide attempt over the past year	NA
Park et al. [45]	2020	Tinnitus and Its Association with Mental Health and Health-Related Quality of Life in an Older Population: A Nationwide Cross-Sectional Study	Korea	General over 60s	5129	1402	Rated ‘annoying’ by 605 participants	For the past executive 12 months, have you ever had ringing, roaring, or buzzing in your ears?	Survey questions: normal, tolerable tinnitus, and annoying tinnitus identified by asking participants with tinnitus. How much do these sounds create annoyance in your life?	Have you ever thought about committing suicide within 12 months?	NA
Yu et al. [44]	2019	Association between menstrual cycle irregularity and tinnitus: a nationwide population-based study	Korea	General, premenopausal Korean women	4633	934	Rated ‘annoying’ by 605 participants	For the past executive 12 months, have you ever had ringing, roaring, or buzzing in your ears?	How much do these sounds create annoyance in your life?	Have you ever thought about committing suicide within the last 12 months?	NA

**Table 3:** Case studies.

Author	Date	Title	Country	Population	Sample Size	Participants with Tinnitus (n)	Tinnitus Severity	Method/Question Used to Identify Tinnitus	Method/Question Used to Identify Bothersome Tinnitus	Method/Question Used to Identify Suicide/Suicidal Ideation	Method of Suicide
Chang and Wu [50]	2012	Serotonin-Norepinephrine Reuptake Inhibitor Treatment for Tinnitus and Depression	Taiwan	Male with bilateral tinnitus	1	1	Severe- interfering with mood, sleep, and psychosocial functioning	Not reported	Not reported	Not reported	No suicide—ideation reported
Da Silva Souza et al. [51]	2016	Effects of Transcranial Direct Current Stimulation in Chronic Tinnitus Treatment: Case Study	Brazil	Female with bilateral tinnitus	1	1	Not reported	Acuphenometry, VAS	Acuphenometry, VAS	Not reported	No suicide—patient reported ideation
Dijkstra et al. [53]	2018	Effective deep brain stimulation of intractable tinnitus: A case study	Netherlands	Female tinnitus patient	1	1	Intractable tinnitus causing severe suffering	Tinnitus Handicap Inventory, Tinnitus Functional Index	Tinnitus Handicap Inventory, Tinnitus Functional Index	Hamilton Depression Rating Scale	Previous suicide attempt with autointoxication
Ensink et al. [41]	2003	Treatment for Severe Palatoclonus by Occlusion of the Eustachian Tube	Netherlands	Male child with tinnitus	1	1	Intense, loud, severely interfering with sleep	Tinnitus heard by examiner by bringing own ear at a distance of approximately 20 to 30 cm to the left ear of the patient	Not reported	Expressed suicide wish from patient	No suicide—ideation reported
Frankenburg and Hegarty [54]	1994	Tinnitus, Psychosis, and Suicide	USA	Tinnitus patients	4	4	Reported as unrelenting, tiresome	Not reported	Not reported	Expressed suicide wishes from patients. One patient attempted suicide	Not reported
Joshi and Sharma [55]	2012	A Case of Asenapine-Induced Tinnitus	USA	Female patient	1	1	Distressing	Not reported	Not reported	Not reported	No suicide—ideation reported. Suicidal ideation
Lewis and Stephens [56]	1995	Parasuicide and tinnitus	UK	Patients admitted for overdose to Poisons Unit	184	3	Not reported	Do you suffer from tinnitus, that is, noise(s) in your ears or head? (yes, no). Please describe the tinnitus (pulsatile, buzzing, whistling, hissing, other)	What influence did the tinnitus have on your decision to take an overdose? (none, it contributed, the main reason)	Patients were admitted for previous overdose	Self-harm /Attempted suicide by overdose
Lewis, Stephens, and Huws [57]	1992	Suicide in tinnitus sufferers	UK	Clinical tinnitus patients, and one additional account	6	6	Severe	Various	Not reported	Reported suicides in clinic patients	Hanging, attempted overdose, of alcohol and drugs, overdose of antidepressants, overdose of pain medication, suffocation
Lewis, Stephens, and McKenna [42]	1994	Tinnitus and suicide	UK	Clinical tinnitus patients	28	28	Not reported	Tinnitus duration: Years and months Tinnitus ear: right/left/both/head Nature of tinnitus: pulsatile/whistling/buzzing/hissing/other	Not reported	Clinics required to report on patients who die by suicide	Self-poisoning, firearm, suffocation, hanging, drowning
Pridmore et al. [58]	2012	Tinnitus and Suicide: Recent Cases on the Public Record Give Cause for Reconsideration	USA	4 cases assessed by a coroner over 10 years- found in newspaper articles and online	4	4	Not reported	Not reported	Not reported	Newspaper and Web search for articles published over the last 10 years	Jumped from height. Hanging. Firearms. Self-stabbing
Fox-Thomas [52]	2016	Suicidal Ideation Among Patients with Chronic Tinnitus	USA	Clinical records which included the Tinnitus Reaction Questionnaire (TRQ)	200	200	Not significant, significant, mild, moderate, severe, profound	TRQ in clinical record	Using a 5-point Likert scale (0–4), patients rated tinnitus distress for 26 items ranging from “not at all” to “almost all of the time”	My tinnitus has led me to think about suicide (#24 on TRQ). how tinnitus has affected you over the past week	NA
Sisler et al. [59]	2015	Self-Decapitation Attempt Attributed to Tinnitus and Oral Corticosteroids	USA	Clinical patient	1	1	Severe tinnitus	Case report	Case report	Case report	NA—attempted by self-poisoning and self-laceration

TRQ = Tinnitus Reactions Questionnaire. VAS = Visual Analogue Scale.

**Table 4:** Observational studies.

Author	Date	Title	Country	Population	Sample Size	Participants with Tinnitus (n)	Tinnitus Severity	Method/Question Used to Identify Tinnitus	Method/Question Used to Identify Bothersome Tinnitus	Method/Question Used to Identify Suicide/Suicidal Ideation	Method of Suicide
Aazh and Moore [61]	2018	Thoughts about Suicide and Self-Harm in Patients with Tinnitus and Hyperacusis	UK	Clinical tinnitus patients—specialist clinic for patients seeking help with tinnitus	150	144	32 mild, 42 moderate, 54 severe	Questionnaire	Tinnitus Handicap Inventory, VAS	Question 9 on PHQ9	No suicide—study measured ideation
Lugo et al. [64]	2019	Sex-Specific Association of Tinnitus With Suicide Attempts	Sweden	Adults from Stockholm County (Sweden)	71,542	16,066 (any tinnitus), of which 1995 were severe	“No; Yes (moderate problem); Yes (severe problem”)	“Do you have any of the following health problems or symptoms?” …tinnitus….	Not reported	“Have you ever tried to take your own life?”	NA
Aazh et al. [62]	2019	Parental Mental Illness in Childhood as a Risk Factor for Suicidal and Self-Harm Ideations in Adults Seeking Help for Tinnitus and/or Hyperacusis	UK	Patients at Tinnitus and Hyperacusis Therapy Specialist Clinic	292	286	Mild, moderate, severe	Tinnitus Handicap Inventory	Tinnitus Handicap Inventory	Question 9 on PHQ99.	No suicide—ideation reported
Cheng et al. [65]	2023	Tinnitus and risk of attempted suicide: A one year follow-up study	Taiwan	Patients aged ≥20 years old who received a first-time diagnosis of tinnitus and no history of suicide for 3 years before tinnitus onset. Matched controls with no tinnitus	386,055	Subjective Any—includes codes for tinnitus, unspecified	Not reported	At least two outpatient medical claims with a diagnosis of tinnitus filed by an otorhinolaryngologist or a neurologist	none	None–not recorded in ICD codes. Whether or not a sampled patient had received a diagnosis of suicide attempt during one-year follow-up. ICD codes	NA
Chen et al. [63]	2023	The Comorbidity of Depression and Anxiety Symptoms in Tinnitus Sufferers: A Network Analysis	China	Tinnitus sufferers aged over 11 years followed up at the tinnitus outpatient department	566	566	0–10 VAS; Minimal = 0–3, mild = 4–6), Severe = 7–10	Not reported	VAS	Question 9 on PHQ9	NA

ICD = International Classification of Disease. NA = not applicable. PHQ9 = patient Health Questionnaire 9. VAS = Visual Analogue Scale.

**Table 5:** Consensus definitions of terms commonly used in suicide research [11].

Suicide	Intentionally ending one’s own life
Suicidal behaviour	Behaviours that may result in ending one’s life, whether fatal or not. This term excludes suicidal ideation
Suicidal ideation	Any thoughts about ending one’s own life. May be active, with a clear plan for suicide, or passive, with thoughts about wishing to die
Suicide attempt	Self-injurious non-fatal behaviour with inferred or actual intent to die
Self-harm	Self-injurious behaviours with or without intent to die. Does not distinguish between suicide attempt and non-suicidal self-injury
Non-suicidal self-injury	Self-injurious behaviours without any intent to die

## Data Availability

This is a literature review of previously published records and all records are in the public domain. Summaries of the included records are provided on Supplemental Materials.

## Supplementary Materials

The following supporting information can be downloaded at: https://www.mdpi.com/article/10.3390/brainsci13101496/s1, Table S1. Epidemiological studies; Table S2. Observational studies; Table S3. Case studies.
