# Supplementary material for: Tinnitus, Suicide, and Suicidal Ideation: A Scoping Review of Primary Research
Source: Brain Sci. 2023 Oct 23;13(10):1496. doi: 10.3390/brainsci13101496 (PMC10605905; doi:10.3390/brainsci13101496)
Supplement: Supplementary file 1 [file brainsci-13-01496-s001.zip › brainsci-2616969-supplementary.pdf]

Table S1. Epidemiological studies

Author	Date	Title	Country	Population	Sample size	Participants with tinnitus (n)	Tinnitus severity	Method/question used to identify tinnitus	Method/question used to identify bothersome tinnitus	Method/question used to identify suicide/suicidal ideation	Method of suicide
Han et al	2018	Tinnitus, depression, and suicidal ideation in adults: A nationally representative general population sample	Korea	Respondents of tinnitus evaluation questionnaires of the KNHANES aged 19+	28,930	6391	Mild, moderate and severe (no disturbance, annoying and very annoying)	During the past year, did you ever hear a sound (buzzing, hissing, ringing, humming, roaring, machinery noise) originating in your ear? (yes,no, cannot remember)	How much did the sound originating in your ear disturb your daily life? (no, annoying, very annoying level causing sleep disturbance).	Within the past year, have you ever seriously considered attempting suicide? (yes or no)	No suicide - ideation reported
Martz et al	2018	Tinnitus, Depression, Anxiety, and Suicide in Recent Veterans: A Retrospective Analysis	USA	Veterans who separated from service and who were not killed in action.	769,934	116,358 (15%)	Not reported	NA- diagnosed with tinnitus	Not reported	Not reported	ICD-10 codes were used to identify cause of death.
Kim et al	2018	Association between tinnitus and mental health among Korean adolescents: The Korea National Health and Nutrition Examinat	Korea	Adolescent respondents of the KNHANES V	1587	286	no problem, bothering, having trouble sleeping	In the past 12 months, have you been bothered by buzzing in your ears? (yes, no)	How much of a problem is the ringing in your ears? (no problem, bothering, having trouble sleeping).	'Have you ever thought of committing suicide in the last year? (yes, no)	No suicide-ideation reported

		ion Survey									
Seo et al	2015	Relationship between tinnitus and suicidal behaviour in Korean men and women: a cross-sectional study	Korea	Korean individuals who participated in 2010 to 2012 Korean National Health and Nutrition Examination Survey (KNHANES).	17446	3949 21.4%	not annoying/annoying; irritating; severely annoying and causes sleep problems	'Within the past year, did you ever hear a sound (buzzing, hissing, ringing, humming, roaring, machinery noise) originating in your ear?'	'How severe is this noise in your daily life?	In the last 12 months, did you think about committing suicide?	NA
Park et al	2020	Psychiatric Distress as a Common Risk Factor for Tinnitus and Joint Pain: A National Population-Based Survey	Korea	General over 50s	9032	2413	classified as none, not annoying, annoying, and severely annoying	single question	single question: none, not annoying, annoying, severely annoying.	positive answer to the question about suicidal ideation and suicide attempt over the past year	NA
Park et al	2020a	Tinnitus and Its Association With Mental Health and Health-Related Quality of Life in an Older Population: A Nationwide Cross-Sectional Study	Korea	General over 60s	5129	1402	Rated 'annoying' by 605 participants	"For the past executive 12 months, have you ever had ringing, roaring, or buzzing in your ears?"	Survey questions: normal, tolerable tinnitus, and annoying tinnitus identified by asking participants with tinnitus "How much do these sounds create annoyance in your life?."	"Have you ever thought about committing suicide within 12 months?"	NA

Yu et al	2019	Association between menstrual cycle irregularity and tinnitus: a nationwide population-based study	Korea	General, premenopausal Korean women	4633	934	Rated 'annoying' by 605 participants	"For the past executive 12 months, have you ever had ringing, roaring, or buzzing in your ears?"	"How much do these sounds create annoyance in your life?"	"Have you ever thought about committing suicide within the last 12 months?"	NA

Table S2. Observational studies

AUTHOR	DATE	TITLE	COUNTRY	POPULATION	SAMPLE SIZE	PARTICIPANTS WITH TINNITUS (N)	TINNITUS SEVERITY	METHOD/QUESTION USED TO IDENTIFY TINNITUS	METHOD/QUESTION USED TO IDENTIFY BOTHERSOME TINNITUS	METHOD/QUESTION USED TO IDENTIFY SUICIDE/SUICIDAL IDEATION	METHOD OF SUICIDE
Aazh & Moore	2018	Thoughts about Suicide and Self-Harm in Patients with Tinnitus and Hyperacusis	UK	Clinical tinnitus patients - specialist clinic for patients seeking help with tinnitus	150	144	32 pps - mild, 42 pps - moderate, 54 pps - severe	Tinnitus Handicap Inventory, The Visual Analog Scale (VAS)	Tinnitus Handicap Inventory, VAS	Question 9 on PHQ9	No suicide– study measured ideation
Lugo et al	2019	Sex-Specific Association of Tinnitus With Suicide Attempts	Sweden	Adults from Stockholm County (Sweden)	71,542	16 066 (any tinnitus), of which 1995 were severe	"No; Yes (moderate problem); Yes (severe problem").	"Do you have any of the following health problems or symptoms?" …tinnitus….	Not reported	"Have you ever tried to take your own life?"	NA
Aazh et al	2019	Parental Mental Illness in Childhood as a Risk	UK	Patients at Tinnitus and Hyperacusis	292	286	Mild, moderate, severe	Tinnitus Handicap Inventory	Tinnitus Handicap Inventory.	Question 9 on PHQ9 9.	No suicide - ideation

		Factor for Suicidal and Self-Harm Ideations in Adults Seeking Help for Tinnitus and/or Hyperacusis		Therapy Specialist Clinic							reported
Cheng et al	2023	Tinnitus and risk of attempted suicide: A one year follow-up study	Taiwan	Patients aged ≥20 years old who received a first-time diagnosis of tinnitus and no history of suicide for 3 years before tinnitus onset. Matched controls with no tinnitus.	386,055	subjective<br><br>Any - includes codes for tinnitus, unspecified.	Not reported	At least two outpatient medical claims with a diagnosis of tinnitus filed by an otorhinolaryngologist or a neurologist	none	none-not recorded in ICD codes. whether or not a sampled patient had received a diagnosis of suicide attempt during one-year follow-up. ICD codes	NA
Chen et al	2023	The Comorbidity of Depression and Anxiety Symptoms in Tinnitus Sufferers: A Network Analysis	China	Tinnitus sufferers aged over 11 years followed up at the tinnitus outpatient department in Chinese PLA General Hospital	566	566	0-10 VAS; Minimal = 0–3, mild = 4–6), Severe = 7–10	Not reported	VAS	Question 9 on PHQ9	NA

NA = not applicable, PHQ9 = Patient Health Questionnaire 9, VAS = Visual Analogue Scale.

Table S3. Case studies

Author	Date	Title	Country	Population	Sample size	Participants with tinnitus (n)	Tinnitus severity	Method/question used to identify tinnitus	Method/ question used to identify bothersome tinnitus	Method/ question used to identify suicide/suicidal ideation	Method of suicide
Chang & Wu	2012	Serotonin-Norepinephrine Reuptake Inhibitor Treatment for Tinnitus and Depression	Taiwan	Male with bilateral tinnitus	1	1	Severe - interfering with mood, sleep and psychosocial functioning	Not reported	Not reported	Not reported	No suicide - ideation reported
Da Silva Souza et al	2016	Effects of Transcranial Direct Current Stimulation in Chronic Tinnitus Treatment: Case Study	Brazil	Female with bilateral tinnitus	1	1	Not reported	acuphenometry, VAS	acuphenometry, VAS	Not reported	No suicide - patient reported ideation
Dijkstra et al	2018	Effective deep brain stimulation of intractable tinnitus: A case study	Netherlands	Female tinnitus patient	1	1	Intractable tinnitus causing severe suffering	Tinnitus Handicap Inventory, Tinnitus Functional Index	Tinnitus Handicap Inventory, Tinnitus Functional Index	Hamilton Depression Rating Scale	Previous suicide attempt with autointoxication
Ensink et al	2003	Treatment for Severe Palatoclonus by Occlusion of the	Netherlands	Male child with tinnitus	1	1	Intense, loud, severely interfering with sleep	Tinnitus heard by examiner by bringing own ear at a distance of approximately 20 to 30 cm to the left ear of the patient.	Not reported	Expressed suicide wish from patient	No suicide - ideation reported

		Eustachian Tube									
Frankenburg & Hegarty	1994	Tinnitus, Psychosis, and Suicide	USA	Tinnitus patients	4	4	Reported as unrelenting, tiresome	Not reported	Not reported	Expressed suicide wishes from patients. One patient attempted suicide.	Not reported
Joshi & Sharma	2012	A Case of Asenapine-Induced Tinnitus	USA	Female patient	1	1	Distressing	Not reported	Not reported	Not reported	No suicide - ideation reported. Suicidal ideation.
Lewis & Stephens	1995	Parasuicide and tinnitus	UK	Patients admitted for overdose to Poisons Unit Aug-Oct 1993	184	3	Not reported	Do you suffer from tinnitus, that is, noise(s) in your ears or head? (yes,no). Please describe the tinnitus (pulsatile, buzzing, whistling, hissing, other).	What influence did the tinnitus have on your decision to take an overdose? (none, it contributed, the main reason).	Patients were admitted for previous overdose.	Self-harm /Attempted suicide by overdose.
Lewis, Stephens and Huws	1992	Suicide in tinnitus sufferers	UK	Clinical tinnitus patients, and one additional account	6	6	Severe	Various	Not reported	Reported suicides in clinic patients	Hanging, attempted overdose, of alcohol and drugs, overdose of antidepressants, overdose of pain medication, suffocation.

Lewis, Stephens and McKenna	1994	Tinnitus and suicide	UK	Clinical tinnitus patients	28	28	Not reported	Tinnitus duration: Years and months Tinnitus ear: right/left/both/head Nature of tinnitus: pulsatile/whistling/buzzing/hissing/other	Not reported	Clinics required to report on patients who die by suicide.	Self-poisoning, firearm, suffocation, hanging, drowning.
Pridmore et al	2012	Tinnitus and Suicide: Recent Cases on the Public Record Give Cause for Reconsideration	USA	4 cases assessed by a coroner over 10 years- found in newspaper articles and online	4	4	NR	Not reported	Not reported	Newspaper and Web search for articles published over the last 10 years	Jumped from height. Hanging. Firearms. Self-stabbing.
Fox-Thomas	2016	Suicidal Ideation Among Patients with Chronic Tinnitus	USA	Clinical records which included the Tinnitus Reaction Questionnaire (TRQ)	200	200	Not significant, significant, mild, moderate, severe, profound	TRQ in clinical record	Using a 5-point Likert scale (0-4), patients rated tinnitus distress for 26 items ranging from "not at all" to "almost all of the time."	"My tinnitus has led me to think about suicide" (#24 on TRQ). how tinnitus has affected you over the past week."	NA
Sisler et al	2015	Self-Decapitation Attempt Attributed to Tinnitus and Oral Corticosteroids	USA	clinical patient	1	1	severe tinnitus	case report	case report	case report	NA- attempted by self-poisoning and self-laceration

ICD= International Classification of Disease.
